# Early adversity and sexual diversity: the importance of self-reported and neurobiological sexual reward sensitivity

**DOI:** 10.1038/s41598-024-58389-w

**Published:** 2024-04-15

**Authors:** Jenna C. Alley, Amy S. McDonnell, Lisa M. Diamond

**Affiliations:** 1 Department of Psychiatry and Biobehavioral Sciences, University of California, 760 Westwood Plaza, Los Angeles, CA 90095 USA; 2 Department of Psychology, University of Utah, Salt Lake City, UT USA

**Keywords:** Life History Theory, Early adversity, Sexual diversity, Sexual risk taking, EEG reward sensitivity, Psychology, Human behaviour

## Abstract

Work shows that sexually-diverse individuals face high rates of early life adversity and in turn increased engagement in behavioral outcomes traditionally associated with adversity, such as sexual risk taking. Recent theoretical work suggests that these associations may be attributable to *heightened sexual reward sensitivity* among adversity-exposed women. We aimed to test these claims using a combination of self-report and EEG measures to test the relationship between early adversity, sexual reward sensitivity (both self-reported and EEG measured) and sexual risk taking in a sexually diverse sample of *cis*-gender women (N = 208) (Mage = 27.17, SD = 6.36). Results showed that childhood SES predicted self-reported sexual reward sensitivity which in turn predicted numbers of male and female sexual partners. In contrast we found that perceived childhood unpredictability predicted neurobiological sexual reward sensitivity as measured by EEG which in turn predicted male sexual partner number. The results presented here provide support for the notion that heightened sexual reward sensitivity may be a pathway through which early life adversity augments future sexual behavior, and underscores the importance of including greater attention to the dynamics of *pleasure and reward* in sexual health promotion.

## Introduction

Approximately 30% of sexual and gender diverse (SGD; often denoted LGBTQ+ or sexual minority) individuals face early adversity, which is almost two times higher than rates seen in heterosexual populations^1^. Research shows that individuals reporting more early adversity, such as childhood abuse or neglect, are more likely to engage in sexual behaviors that increase rates of STD/STI diagnoses and unintended pregnancy^2–6^. Although extensive research has documented these associations, we have yet to understand *the full range of mechanisms through which* individuals with exposure to early adversity show heightened sexual risk taking which is critically important to the development of effective sexual health interventions. The present work addresses this gap in the literature by examining *sexual reward sensitivity* (SRS; i.e., increased cognitive, behavioral, and neurobiological *attention* and *reaction* to sex specific rewards) as a mechanism through which early adversity relates to heightened sexual risk taking^7^.

### Early adversity and sexual risk behavior

Early life adversity, defined in terms of neglect, abuse, and household unpredictability^8^, is associated with a greater likelihood of engaging in sexual risk behaviors such as earlier age of first sexual experience^9–11^, having more sexual partners^5,6,9,10,12–16^, which in turn contributes to the increased risk for pregnancy and STD/STIs^15,17,18^ seen in adversity exposed individuals. While various forms of early adversity such as abuse and neglect have long been established as predictors of risk taking there is also evidence that low socioeconomic status (SES) across life may also be a strong predictor of negative sexual health outcomes by way of increased sexual risk behavior ^19,20^

Further, early adversity has been observed to predict greater engagement in *multiple* types of sexual risk taking^21–23^ including same gender sexual behavior^24^. Disproportionate exposure to early adversity among sexually diverse populations may help to explain such patterns. Numerous studies have found that SGD individuals report disproportionate levels of early adversity^25–28^. Interestingly, past work has found that women who identify as “mostly heterosexual” also report higher rates of early adversity when compared to exclusively heterosexual women^26^ which indicates that the relationship between early adversity and sexual diversity is not restricted to individuals who openly identify as SGD.

Although same-gender behavior differs from other widely studied sexual risk behaviors given that it is not typically considered a health "risk behavior" for women (given that it is associated with less STI/STD risk than other-gender behavior, and no risks for pregnancy) it does however confer significant *social* risks due to the widespread marginalization of SGD populations. Further, individuals who engage in same-gender behavior also tend to report greater overall sexual risk taking, including lack of condom use^29^, engaging in sex at an earlier age and having higher numbers of sexual partners^29–33^. Therefore when aiming to understand the full range of sexual risk—be it social or physical—that could be related to early adversity exposure, it is important for researchers to expand their investigations of early adversity to include a focus on sexual behaviors that do not fit the standard profile of a “sexual risk behavior,” such as women’s same-gender behavior. As reviewed by Diamond and Alley^34^, it is not clear *how* sexually-diverse populations show high rates of early adversity; however, evolutionary-developmental theory can help provide insight.

Life History Theory (LHT) proposes that early environmental experiences are treated by the evolved human brain as “signals” of what the future will be like, and these signals trigger *adaptations* designed to foster survival in that environment. Accelerated sexual maturation and heightened sexual risk behavior are among these adaptations: they increase one’s chances for reproducing in a dangerous environment, where life is short, resources scarce, and parental nurturance cannot be counted on. Hence, the behaviors that are considered “risky” from a modern perspective were adaptive in the human ancestral environment, because they fostered the ultimate survival of that individual in their environment given the perceived and experienced unpredictability and or harshness^35^. Support for this view comes from the extensive body of research^7^ outlined above showing increased sexual risk taking in individuals who have experienced adversity in early life.

As noted by Ellis et al. ^8^ LHT is more successful at explaining *why* early adversity is associated with high-risk sexual behavior than explaining *how* this association unfolds. Specifically, we have yet to understand what specific cognitive-behavioral-developmental mechanisms “nudge” adversity-exposed individuals toward earlier and higher-risk sexual behavior. Most previous research on this question has focused on social mechanisms^7^ such as increased risk taking due to low parental supervision^36–38^. While these social mechanisms are undoubtedly valid, Alley and Diamond^7^ argued that in addition to these processes, early adversity may augment future sexual behavior by way of *heightened sensitivity to sexual rewards* which can motivate sexual risk engagement despite the potential risks associated. Some evidence for such associations comes from work examining the impact of adversity exposure on general reward processing and behavior.

### Previous research on adversity and reward sensitivity

Although no prior work has directly examined associations between early adversity and *sexual* reward sensitivity, previous work has shown that early adversity augments behavioral reward sensitivity, such that adversity exposed individuals show a tendency to pursue immediate versus delayed rewards^39^, prefer immediate versus delayed rewards^40^ and are willing to take greater risk for greater reward^41^. This suggests that early adversity does augment reward sensitivity in a general sense and therefore may do the same to behavior-specific sensitivity.

Reward sensitivity is also mapped on a neurobiological level, allowing for insight into more automatic processes that self-report measures, by definition, cannot capture. Several studies have examined the impact of early adversity on neural substrates of reward processing using fMRI and EEG, though most do so with monetary rather than sexual rewards. These studies have demonstrated changes in reward-related brain activity associated with poor neighborhood quality^42^, family adversity^43^, SES^44^, abuse/neglect^45^, childhood stress^40^, and low parental warmth^46^. However, all of these studies focus on how early adversity can augment general reward sensitivity such as monetary rewards, but given the unique aspects of sexuality and sexual stimuli processing^47^, it is important that we consider sexual reward sensitivity as distinct from general reward sensitivity^7^.

### Sexual reward sensitivity as a distinct construct

It goes without saying that sexual behavior provides pleasure, and individuals seeking such gratification may have to make trade-offs to obtain it (in the modern context, these trade-offs may include reputational concerns, risks for pregnancy, and risks for STI/STD). The pursuit of physical pleasure, which is a strong motivator for sexual behavior^48^, provides a strong motivation to pursue high-risk versus low-risk sexual behavior given that many if not all of the high-risk sexual behaviors result in more pleasure or are more motivated by sexual pleasure than less risky alternatives. For example, while motivations for sex within committed relationships tend to be multifactorial^48–51^ both men and women report that sexual pleasure is a key motivator for engagement in casual sex^49,52^ which facilitates higher sexual partner number.

Accordingly, Alley and Diamond^7^ argue that early adversity may augment future sexual behavior by consistently shifting individuals toward the “pleasure/reward” side of the trade-off, strengthening individuals’ motives for sexual reward even when it comes with risks and costs such as punishment, disease, pregnancy, physical harm, or stigma^7^. This view suggests that we cannot fully understand the link between early adversity and sexual risk behavior without attending to one of the most distinctive characteristics of sexual behavior: physical pleasure.

Further providing support for their claims, heightened sensitivity to sexual reward among adversity-exposed individuals also helps to explain one of the most robust—and perplexing—findings from research on early adversity and sexuality: individuals exposed to early adversity are disproportionately likely to engage in same-gender behavior. Alley and Diamond^7^ argue that for adversity-exposed individuals, same-gender behavior may be similar to other “high risk” sexual behaviors, because it offers high sexual reward (pleasure) while entailing significant risks. Contradictory to the risks for men (potential harm and STD/STIs), same gender behavior risks for women are primarily social. Further, same gender behavior for women is particularly rewarding. For example, extensive research has found that men are more likely than women to reach orgasm (one metric of sexual pleasure) through penile-vaginal intercourse^31,53–55^, especially during casual sexual encounters^49^ and orgasm rates are far higher in women who engage in sex with other women^56^. Hence, if early life adversity amplifies a woman’s responsiveness to sexual reward, then lesbian- and bisexually oriented women exposed to adversity may be more likely to *act on* their same-gender attractions, and heterosexually-oriented women may be more likely to act on their *capacity* for sexual fluidity^57^ by seeking sexual pleasure with both male and female partners^7^.

### Neurobiological sexual reward sensitivity

There is a small EEG literature that assesses neural responses to *sexual* rather than monetary stimuli. For example, Huberman et al.^58^ identified several components of the event-related brain potential (ERP) called the P300 and anterior N270-400 that are sensitive to sexual readiness such that they fluctuate in response to viewing an erect versus flaccid penis. Prause et al.^59^ found changes in an ERP component called the late positive potential (LPP) in response to explicit versus non-explicit sexual images—which varied based on individual differences in number of sexual intercourse partners. These EEG findings support the notion that sexual reward may underlie individuals’ pursuit of high-risk sexual behavior, and that these relationships can be indexed with ERP components. However, no previous research has directly tested whether *early life adversity* is associated with enhanced neural sensitivity to sexual *rewards*. The present study seeks to fill this gap in the literature by quantifying an ERP called the reward positivity (RewP), which is a deflection in the EEG waveform that appears in response to the presentation of reward-related stimuli and is more positive for rewarding stimuli than for non-rewarding stimuli^60^. The present study takes a novel approach to quantifying the RewP in response to sexual compared to non-sexual images as a neurobiological index of sensitivity to sexual reward.

## Current study

The current study uses a combination of self-report and EEG methodology, in a sample of women with diverse sexual identities and experiences, to test associations between early adversity and (1) self-reported preferences for rewarding-but-risk-prone sexual behavior, (2) neurobiological responsiveness to sexual rewards, and (3) actual engagement in high-risk sexual behavior.

### Hypotheses

 

1. Early adversity will indirectly increase sexual risk taking via its direct enhancement of self-reported sexual reward sensitivity.
2. Early adversity will indirectly increase sexual risk taking via  its direct enhancement of neurobiological sexual reward sensitivity.
3. The association between early adversity and increased sexual risk taking will function through both self-reported and neurobiological sexual reward sensitivity when estimated simultaneously.

## Results

Table 1 provides descriptive statistics as well as zero order correlations for all study variables.

**Table 1:** Means, standard deviations, and correlations with confidence intervals.

Variable	*M*	*SD*	1	2	3	4	5	6	7	8	9	10	11
1. Sexual abuse	0.29	0.45											
2. Perceived unpredictability	1.45	0.91	0.17* [0.03, 0.31]										
3. Nonsexual abuse	1.23	1.19	0.22** [0.08, 0.35]	0.58** [0.48, 0.66]									
4. Self-reported SRS	11.46	2.62	− 0.04 [− 0.18, 0.10]	− 0.03 [− 0.16, 0.11]	− 0.06 [− 0.20, 0.08]								
5. Childhood SES	1.36	1.12	− 0.16* [− 0.29, − 0.01]	− 0.45** [− 0.55, − 0.33]	− 0.37** [− 0.49, − 0.25]	− 0.14 [− 0.27, 0.00]							
6. # Female sexual partners	0.44	0.38	− 0.02 [− 0.21, 0.17]	0.04 [− 0.14, 0.23]	0.04 [− 0.15, 0.22]	0.22* [0.04, 0.39]	0.06 [− 0.13, 0.24]						
7. # Male sexual partners	2.02	1.16	0.02 [− 0.14, 0.17]	0.23** [0.08, 0.36]	0.13 [− 0.01, 0.28]	0.20** [0.06, 0.34]	− 0.16* [− 0.30, − 0.01]	0.30** [0.11, 0.46]					
8. Earliest debut	16.60	3.37	− 0.14 [− 0.29, 0.01]	− 0.18* [− 0.32, − 0.04]	− 0.20** [− 0.33, − 0.05]	− 0.02 [− 0.17, 0.12]	0.09 [− 0.06, 0.23]	− 0.08 [− 0.26, 0.11]	− 0.23** [− 0.36, − 0.08]				
9. Safe sex behavior	1.72	0.79	0.02 [− 0.12, 0.17]	− 0.06 [− 0.20, 0.08]	0.02 [− 0.12, 0.16]	− 0.09 [− 0.22, 0.05]	0.06 [− 0.08, 0.20]	− 0.08 [− 0.26, 0.10]	− 0.06 [− 0.21, 0.08]	− 0.02 [− 0.16, 0.13]			
10. Image type	1.94	0.47	− 0.20 [− 0.47, 0.09]	0.20 [− 0.09, 0.45]	0.17 [− 0.12, 0.43]	0.22 [− 0.06, 0.47]	− 0.16 [− 0.42, 0.12]	0.42* [0.08, 0.68]	− 0.00 [− 0.30, 0.30]	0.16 [− 0.14, 0.43]	0.17 [− 0.11, 0.43]		
11. Image arousal	5.83	1.98	− 0.36* [− 0.60, − 0.06]	0.23 [− 0.07, 0.49]	0.13 [− 0.17, 0.41]	0.24 [− 0.06, 0.50]	− 0.08 [− 0.37, 0.22]	− 0.16 [− 0.51, 0.22]	− 0.08 [− 0.38, 0.24]	− 0.02 [− 0.32, 0.29]	0.05 [− 0.25, 0.34]	0.27 [− 0.04, 0.52]	
12. Neurobiological SRS	4.60	2.92	0.06 [− 0.20, 0.31]	− 0.32* [− 0.52, − 0.07]	− 0.13 [− 0.37, 0.12]	0.26* [0.01, 0.47]	0.23 [− 0.02, 0.45]	− 0.09 [− 0.40, 0.24]	− 0.08 [− 0.33, 0.19]	0.16 [− 0.11, 0.40]	0.12 [− 0.13, 0.36]	− 0.10 [− 0.37, 0.19]	− 0.01 [− 0.31, 0.28]

*M* and *SD* are used to represent mean and standard deviation, respectively. Values in square brackets indicate the 95% confidence interval for each correlation. The confidence interval is a plausible range of population correlations that could have caused the sample correlation (Cumming, 2014). *p < 0.05. **p < 0.01.

### ***Self-reported SRS (***Table 2***)***

**Table 2:** Standardized path estimates for Hypotheses 1.

Predictor	Self-reported SRS β (SE)	# Female sexual partner β (SE)	# Male sexual partner β (SE)	Safe sex behavior β (SE)	Earliest debut β (SE)
Non-sexual abuse	− 0.10 (0.20)	–	–	–	–
Perceived unpredictability	− 0.06 (0.26)	–	–	–	–
Sexual Abuse	− 0.03 (0.44)	–	–	–	–
Childhood SES	− 0.20 (0.19)*	–	–	–	–
Self-reported SRS	–	0.24 (0.01)**	0.22 (0.03)**	− 0.09 (0.02)	− 0.02 (0.10)

*p < 0.05; **p < 0.01.

#### Hypothesis 1

**Figure 1:**
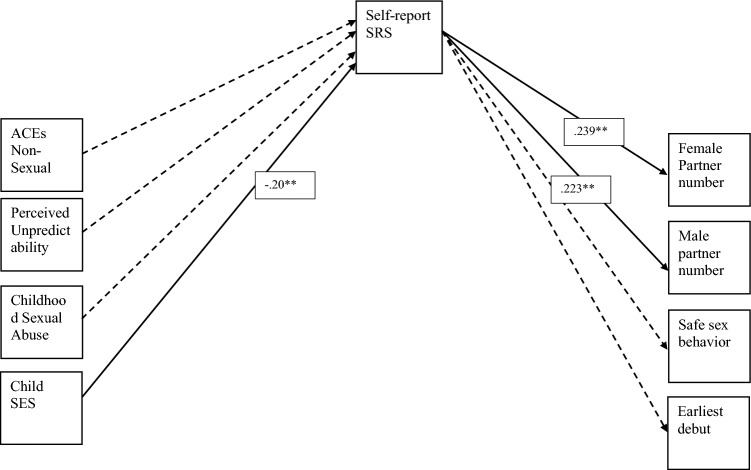
Path model for Hypothesis 1. (Solid black paths are significant paths and dashed black are modeled but non-significant).

Hypothesis 1 (Table 2, Fig. 1) estimates all relevant self-report paths, and predicts that early adversity will indirectly predict sexual risk taking by way of self-reported SRS. The fit of this model is good (χ^2^(16) = 20.030, p = 0.219, CFI = 0.978, RMSEA = 0.035). For significant results, we found that lower childhood perceived socioeconomic status uniquely predicted greater self-reported SRS (*β* = − 0.20, *p* = 0.01) even when controlling for the effects of all other forms of early adversity. We also found that self-reported SRS significantly predicted greater numbers of male sexual partners (*β* = 0.22, *p* = 0.002) and greater numbers of female sexual partners (*β* = 0.24, *p* = 0.004). All other paths were non-significant. Specifically nonsexual abuse (*β* = − 0.10, *p* = 0.27), sexual abuse (*β* = − 0.03, *p* = 0.67) and perceived unpredictability (*β* = − 0.06, *p* = 0.53) did not significantly predict self-reported SRS. Lastly, self-reported SRS did not significantly predict safe sex behavior (*β* = − 0.08, *p* = 0.21) or sexual debut age (*β* = − 0.02, *p* = 0.78).

### ***Automatic, neurobiological SRS (***Table 3***)***

**Table 3:** Standardized path estimates for Hypotheses 2.

Predictor	Neurobiological SRS β (SE)	# Female sexual partner β (SE)	# Male sexual partner β (SE)	Safe sex behavior β (SE)	Earliest debut β (SE)
Non-sexual abuse	0.13 (0.38)	–	–	–	–
Perceived unpredictability	− 0.58 (0.57)**	–	–	–	–
Sexual Abuse	0.28 (10.06)	–	–	–	–
Childhood SES	0.15 (0.32)	–	–	–	–
Image Type	0.04 (0.76)	–	–	–	–
Image Arousal	0.34 (0.29)	–	–	–	–
Neurobiological SRS	–	− 0.18 (0.02)	− 0.25 (0.04)**	0.12 (0.03)	0.25 (0.15)

*p < 0.05; **p < 0.01.

#### Hypotheses 2

**Figure 2:**
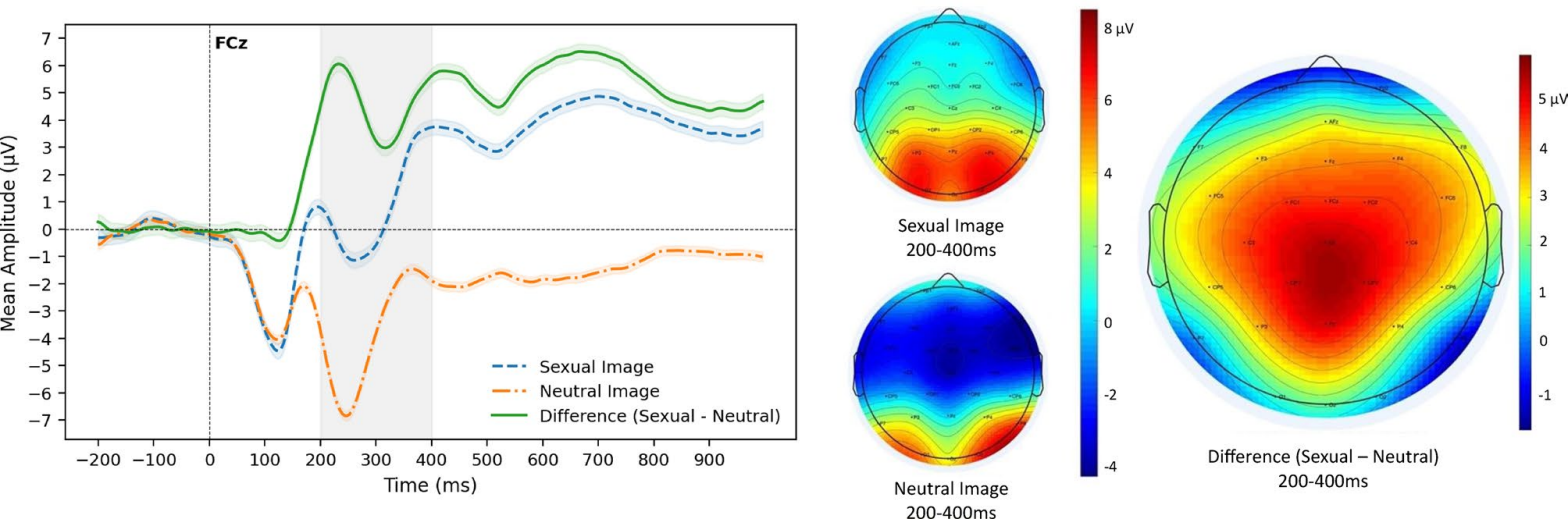
ERP waveforms and scalp maps, averaged across all participants. Left: Grand average conditional (sexual and neutral) waveforms and the difference waveform (Sexual–Neutral) at electrode FCz. Right: Grand average scalp maps depicting response to the two feedback conditions (sexual and neutral) and the difference scalp map (Sexual–Neutral) from 200 to 400 ms post-feedback presentation.

To explore neurobiological sensitivity to sexual reward, we quantified the mean amplitude of the Sexual–Neutral difference waveform within the typical RewP window of 200–400 ms post-image presentation. As expected, the waveform in response to sexual images (*M* = 0.810 µV, SD = 6.03) was significantly greater (more positive) than the waveform in response to neutral images (M = − 3.79 µV, SD = 4.78), as demonstrated with a paired samples t-test (*t*(63) = 12.50, *p* < 0.001; Fig. 2). Mean amplitude of the difference waveform averaged across all participants was 4.60 µV (SD = 2.92).

**Figure 3:**
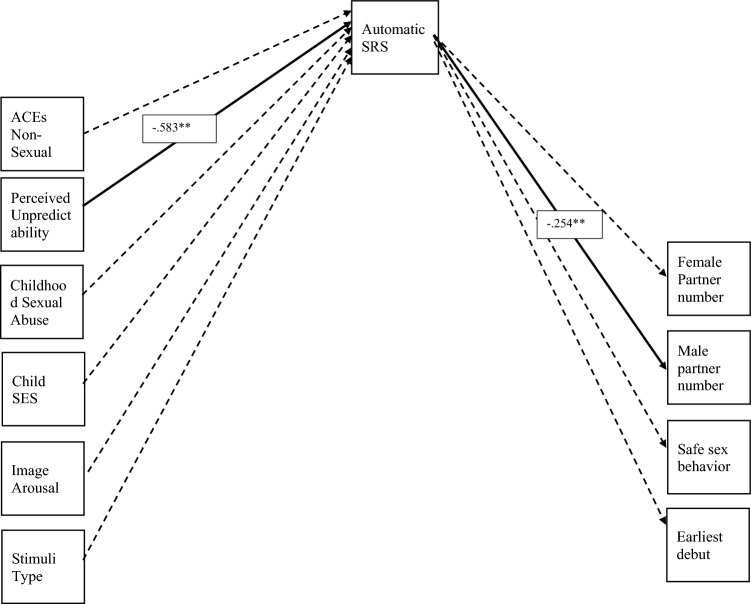
Path model for Hypothesis 2 (Solid black paths are significant paths and dashed black are modeled but non-significant).

Hypothesis 2 (Table 3, Fig. 3) predicted that early adversity would indirectly predict sexual risk taking by way of neural correlates of SRS. We ran the same models as outlined above using mean amplitude of the difference waveform as a neural index of neurobiological SRS. The fit of this specific model is good (χ^2^(24) = 30.441, p = 0.171, CFI = 0.967, RMSEA = 0.036). Contrary to our hypothesis, we found that perceived unpredictability was negatively associated with neurobiological SRS (*β* = − 0.58, *p* = 0.001), suggesting that those with less household unpredictability exhibited greater neurobiological SRS values (i.e., greater amplitude of the difference wave). We also found that this amplitude was significantly associated with number of male sexual partners (*β* = − 0.25, *p* = 0.008). For non-significant results we find that nonsexual abuse (*β* = 0.13 *p* = 0.36), sexual abuse (*β* = 0.28 *p* = 0.07), childhood SES (*β* = 0.15 *p* = 0.17), image arousal (*β* = 0.34 *p* = 0.07), and image type (*β* = 0.04 *p* = 0.79) does not significantly predict neurobiological SRS. Further, we find that neurobiological SRS does not significantly predict female sexual partner number (*β* = − 0.17 *p* = 0.17), safe sex behavior (*β* = 0.12 *p* = 0.28) or sexual debut age (*β* = 0.24 *p* = 0.07).

#### Hypotheses 3

For this hypothesis we simply ran the SEM for hypothesis 1 and 2 estimating all individuals paths simultaneously. However, upon fitting the model we saw that fit statistics were reflecting problems with the data showing CFI values of 1.00 and RMSEA values of 0.000 which is not accurate given that the model was not fully saturated. After probing the data, we concluded that the sample size is too small to be adequately powered for such an analysis and therefore we do not report or interpret the results.

## Discussion

In line with the overarching study aim, our findings provide support for the notion that heightened sexual reward sensitivity may be a pathway through which early life adversity augments future sexual risk behavior. The present research is the first to empirically test and support claims made by Alley and Diamond^7^ as to the importance of sexual reward sensitivity in adversity exposed individuals, providing important preliminary data needed for future research to further our understanding of sexual reward sensitivity and risk taking.

Our results provided support for our first hypothesis regarding self-reported reward sensitivity. We found that women who reported lower childhood SES also self-reported greater responsiveness to sexual rewards even after controlling for all other forms of measured early adversity. In turn, these women reported greater numbers of male and female sexual partners. These results directly support claims made in Alley and Diamond^7^ and suggest that early adversity does in fact have an impact on how one approaches sexual rewards which in turn impacts both social and physical sexual risks upregulating engagement in sex with both same and other gender sexual partners. The results also provide insight into the association between early adversity exposure and sexual orientation^25–28^. Specifically these findings when taken in consideration with evolutionary theory articulated above and in Alley and Diamond^7^ suggests that early adversity does not directly change the ways in which someone sexually identifies (heterosexual vs. gay). But its more likely that early adversity experiences augment the motivation for sexual rewards which impacts both same and other gender sexual behavior that in turn may inform the ways in which one identifies.

Results from our second hypothesis pertaining to neurobiological sexual reward sensitivity are far more complex and provide interesting insight into the claims made by Alley and Diamond^7^ and offer very interesting avenues for further research. Results revealed that women who reported experiencing greater childhood unpredictability showed a *smaller* mean difference between the sexual and neutral images (i.e., smaller difference wave amplitude). This is contradictory to hypothesized, as we expected that greater early adversity would contribute to *greater* differences between the neural response to neutral and sexual images (i.e., greater difference wave amplitude). Instead, results show that those with a smaller difference between the sexual and neutral images (i.e., smaller difference wave amplitude) had higher numbers of male sexual partners which again is contradictory given that we expected that those with greater mean difference wave would engage in more risk taking. While the direction of some associations are not as expected, the pattern of associations are. Specifically, Alley and Diamond^7^ hypothesize that early adversity augments reward processing which in turn increases sexual risk behavior, which we do in fact find here. Specifically, we find that early adversity is associated with smaller mean differences in neurobiological processing of sexual and neutral images and in turn those with smaller mean differences have higher numbers of male sexual partners (a well-studied sexual risk behavior). So while the pattern of association is in line with what was hypothesized, the values are perplexing. Given the extensive evidence for the association between early adversity and sexual risk taking [e.g.^6,10,14,16,21,22,33^] as well as growing evidence pertaining to reward sensitivity and general^61,62^ as well as sexual risk taking^63^ we find it unlikely that our results suggest that early adversity predicts decreased reward sensitivity and in turn less risk taking, but it is more likely that factors associated with our EEG task are impacting the ERP results and in turn SEM associations.

There are a few factors that could be contributing to these perplexing findings. First, this study utilized a novel EEG task designed to measure sexual reward sensitivity (the Sexy Doors Task). In designing this task, we followed the same protocol as the classic Doors Task commonly used to elicit the RewP^60^. However, in the Sexy Doors Task, participants were shown more complex visual scenes (sexual versus neutral images) when compared to simple monetary feedback signals (green versus red arrow) as in the classic Doors Task. It is possible that complex image presentation elicits a co-occurring ERP component called the N2^64^, which may contaminate the RewP component of interest (see Supplementary Materials section “Additional ERP component discussion” for more information). Therefore, the ERP difference wave utilized in our analyses may best be operationalized as simply the difference between neural response to sexual and neutral rewards within the typical RewP window rather than a RewP itself^65^. Despite this nomenclature debate, we still see a more positive conditional waveform in response to sexual images when compared to neutral images, making our difference wave (Sexual–Neutral) positively-valanced and within the time window of the brain’s response to reward, as expected (see Fig. 2). However, the novelty of this task could be a potential reason why the results are not as clear and expected as hypothesized and difficult to map onto prior research.

Another potential factor is that behaviors such as pornography consumption (which are more common among those whose sexual behavior shows characteristics of a “fast” life history strategy)^66^ can have an impact on neurobiological processing of sexual images given familiarity and or comfort with viewing. Specifically, given that there is evidence that sexual experience can have an impact on ERPs when viewing sexual images^59^ and that individuals willing to sign up for sexuality studies generally have higher sexual experience and more positive sexual attitudes, it is possible that our waveform is reflecting the increased sexual experience and comfort of our sample and therefore our sample may simply be *less* responsive to such explicit images. Future work should consider measuring and controlling for such experiences such as porn usage and comfort with sexual imagery. While we cannot make any substantial claims, there is potential that the images we used, the newly designed task and the sample could have simply impacted our ERP waveforms and therefore the interpretation of associations in our SEM models.

Because of the novelty of our work and recent discussion in the EEG literature regarding the overlap between various ERP components within the typical RewP time window^64,65^, we interpret our results with caution. However, our results do provide a solid foundation for future EEG research on the consequences of early adversity for neurobiological responsivity to sexual reward and pleasure.

### Limitations and future directions

As noted earlier, an important limitation to the present study is the inability to rule out the possibility that the relative *novelty* of sexual stimuli may complicate the EEG findings. Given the contemporary availability of sexual images on the internet, and potential associations between high-risk sexual behavior and pornography use, we cannot determine whether familiarity with sexual images played a role in our findings. Future work should seek more comprehensive assessments of individuals’ familiarity with sexual versus nonsexual visual stimuli, and should also consider the inclusion of measures designed to assess the level of novelty and or surprise individuals experience when exposed to sexual stimuli.

Additionally, we designed the cognitive task utilized in this study (the “Sexy Doors Task”) to be based on the classic Doors Task used extensively in prior literature to study neural response to different types of rewarding stimuli, such as money^60^ or food images^65^. In our task, participants choose one of two doors in which behind one door was a sexual image and behind the other door was a neutral image (such as a toaster or towel). We chose these two stimulus categories based on prior work that has found significant differences in ERP components between sexual images and non-human neutral images [e.g.^67–69^]. However, this task design is limited in that is does not control for animacy, such that the sexual images are of human bodies and the neutral images are of objects. This comparison of complex sexual stimuli and more simple control stimuli makes it difficult to draw confident conclusions regarding the role of ERP components in sexual processing, in particular, and not just differences in the neural processing of human forms versus inanimate objects. However, we believe that our study and task design build the groundwork for future studies to include additional control conditions to further disentangle which *aspects* of the sexual images are, in fact, the most rewarding. For example, future work could compare sexual images like the ones used in the present study to non-sexual human images (such as two, clothed adults laying platonically on a bed), rewarding inanimate object images (such as alcohol or food images), or even emotionally-arousing yet non-sexual images (such as pictures of puppies as used by Brown and Cavanagh^64^). Because our work is novel in that it is the first to link early adversity to neurobiological indices of reward processing as well as to integrate sexual stimuli into a gambling paradigm based on the classic Doors Task, we do believe that there are insights to be gained from our task design above and beyond the limitations that may exist.

Another unique aspect and potential limitation of our sample is the fact that all of our participants lived in the Salt Lake City area of Utah. Although we were able to recruit a very sexually diverse sample, due to the predominant religious groups in the area, discussions surrounding pleasure, sexuality and porn are rarely covered in sexual education in Utah; this makes it especially difficult to know whether and how variability in respondents’ exposure to sexual information (and hence their degree of familiarity with sexual images) may have played a role in our findings.

Another potential limitation of the study is that we allowed participants to choose their preferred stimuli type for the EEG task (i.e., women engaging in sex with other women, men engaging in sex with women, or a mix of both). This may be perceived as a limitation only in that not all participants saw the same images. Our goal in allowing this choice was to ensure participants were seeing images that they found maximally rewarding and arousing; however, this variation in stimuli type between participants should be considered when interpreting group-level ERP results. While this is a limitation to be acknowledged, given that the theoretical claims are based on individual differences, our analyses are conducted within subjects and most participants chose the same stimuli type (78% of the sample chose the mixed stimuli). Furthermore, we controlled for stimuli type choice in our models and found no significant associations above and beyond differences in images set choice. Therefore, this potential limitation is unlikely to have a large impact on results and implications.

In advancing this field of research, future scholarship needs to recognize the complexities of trying to disentangle “reward sensitivity” from “risk tolerance.” One of the main tenets of Alley and Diamond’s^7^ argument is that adversity-related sexual reward sensitivity may augment sexual risk taking by amplifying the “rewardingness” of high-risk sexual behaviors. Yet an alternative pathway to the same outcome might be a *dampening* of a woman’s sensitivity to the physical and social *costs* associated with certain sexual rewards. If adversity-exposed women experience the “risk/reward” tradeoff of certain sexual behaviors differently, is this because of a reduction in the “risk” side of the tradeoff or an increase in the “reward” side of the tradeoff? One possibility is that certain types of adversity (such as financial strain) operate more strongly on the “reward” side of the equation whereas others (such as unpredictability) operate more strongly on the “risk” side of the equation. This could provide a potential explanation for the divergent findings across self-reported and neurobiological models. Future research must *integrate* assessments of both risk and reward responsiveness when understanding the impact of early adversity on such processes.

Another important direction for future research involves greater attention to a broader range of sexual risk behaviors than are typically examined in studies of life history strategies, such as same-gender behavior. Alley and Diamond ^7^ argued that if early adversity does augment an individual’s sensitivity to rewards, then we need to devote greater attention to assessing a broader range of behaviors that might be augmented by such a process, such as non-reproductive sexual behaviors that are highly rewarding; this may include not only same-gender behavior, but solitary sexual behavior and other mechanisms for achieving sexual reward. Similarly, in addition to focusing on the health risks of “fast” life history behaviors (such as early sexual debut or sex without contraception), researchers should focus on the social risks of sexual behavior, such as stigma and marginalization (which accompany not only same-gender behavior, but other non-normative sexual practices).

### Implications

While this work has substantial implications in terms of motivating future research as articulated above, these results also provide substantial support for comprehensive sexual education. Specifically, the present results suggest that sexual rewards such as *pleasure* are a core motivation for sexual behavior, regardless of the type of risk. Specifically, we show that an individual’s early experiences with predictability and SES have an impact on the ways in which someone self-reports and processes the potential for sexual rewards which in turn has an impact on their sexual behavior. Therefore, treating sexual rewards such as pleasure, and individuals’ responsiveness to opportunities for pleasure, as *variables* rather than constants may yield superior approaches to sexual health education for both heterosexual and sexually diverse populations. When educating adolescents and adults with a history of adversity about safe sexual practices, it may be important to take account for the fact that they may experience sexual motivations *differently* from other populations, on multiple levels of cognitive processing. Instead of simply asking youths (from both adverse and non-adverse backgrounds) to ignore or suppress their sexual motivations (as is the case with abstinence-based programs, which are relatively ineffective ^70,71^; our research suggests the importance of intervention approaches that forthrightly account for and address individual differences in sexual motivation and sexual reward responsiveness, and can empower youths from diverse backgrounds to actively assess and think through their own particular approach to “reward/risk” tradeoffs. Comprehensive sexual education programs should include discussion of sexual practices that are low in health risks but are highly likely to result in sexual reward and pleasure. In fact, a meta-analysis conducted by Zaneva et al.^72^ shows that sexual health interventions which incorporate considerations of safety and pleasure significantly increase condom use and have positive effects on knowledge-based attitudes surrounding sex.

### Conclusion

Our research provides novel support for the notion that individual differences in sexual reward responsiveness are related to both childhood adversity and adult sexual behavior, supporting the claims made in Alley and Diamond^7^ regarding the importance of sexual reward sensitivity for understanding the unfolding of Life History Strategies. Although future research must replicate these findings and attempt to circumvent some of the limitations of our study design, our findings suggest that disentangling the sources and implications of both self-reported and neurobiological forms of sexual reward sensitivity may play a significant role in understanding the developmental implications of early adversity and the best strategies for promoting sexual health among diverse populations.

## Methods

### Recruitment and eligibility

Participants we recruited by way of paid Facebook advertisements. Interested individuals followed the advertisement to a short Qualtrics survey to assess eligibility. Eligible participants needed to be 18 years or older, live in the greater Salt Lake area in Utah, USA, and be able to take the survey in English. All participants needed to be assigned female at birth and exclusively or primarily identify as a woman. There were no eligibility restrictions in terms of sexual orientation. Further, participants were not eligible for participation in the study if they had a tic or muscular disorder that would introduce muscle artifacts into EEG recordings.

### Procedures and design

All methods and procedures followed the Declaration of Helsinki (1991; p. 1194). All study materials and procedures were approved by the University of Utah Institutional Review Board (IRB). Before completing any survey materials or laboratory sessions all participants provided informed consent.

The study was broken up into two phases of data collection (1) self-report surveys and (2) EEG neurobiological reward sensitivity task in lab. All participants (N = 208) completed the self-report surveys online via Qualtrics prior to their in-lab session. Qualified participants were then scheduled for their in-lab EEG session.

#### Planned missingness design

One of the longstanding weaknesses of previous EEG research is the reliance on small sample sizes due to logistical constrains, making it particularly difficult to detect small effects. A widely-used strategy for addressing such problems is *planned missingness*. We applied this approach by randomly assigning a subset of our participants (*N* = 65) into the EEG (“hard-to-obtain”) condition of the study and administered all other measurements to the full sample of women (*N* = 208). Upon enrollment in the study we used a random number generator for each participant, odd numbers were put in the self-report only group and even numbers were contacted for EEG participation. Interested participants were then scheduled for an in-lab EEG session after completing all online materials. This approach allowed us to use modern missing data techniques to account for this missingness given that it is missing completely at random (MCAR). Please see the Supplementary Materials Section “Planned missingness” for further information regarding the validity of this approach. While our randomization procedures suggest that our EEG data is MCAR, we acknowledge that there could be differences in who agrees to take part in the EEG session. To account for these potential differences, we conducted a series of statistical comparisons to ensure our EEG sample and non-EEG sample are comparable in demographics and study predictors. The result of multiple independent samples t-tests suggest that those enrolled in the EEG portion are not statistically different than those who only enrolled in the self-report portion of the study on the variables examined; age (t_140.359_ = 1.595, p = 0.066), education (*t*_124.163_ = 1.294, p = 0.749), income (*t*_130.651_ = 0.963, p = 0.161), perceived unpredictability (*t*_133.993_ = − 0.637, p = 0.560), sexual abuse (*t*_107.185_ = − 0.696, p = 0.177), childhood SES (*t*_112.795_ = − 0.936, p = 0.097) and nonsexual abuse (*t*_126.302_ = 1.097, p = 0.297).

### Participants

Participants were 208 sexually diverse cis gender women (i.e., individuals who were assigned female at birth and primarily or exclusively identify as a woman) (Mage = 27.17, SD = 6.36) recruited through Facebook. Participants reported a wide distribution of sexual orientations (2.3% Asexual, 20% Bisexual, 28.4% Heterosexual, 8.4% Lesbian, 15.8% Mostly Heterosexual, 6.5% Queer, 1.4% Questioning, 9.3% Pansexual, 3.3% did not identify with the listed options). The sample had a good distribution of income with 25.4% making 25 k or less 40.6% making 25–55 k and 34% making above 55 k. Similarly, there was a good distribution of educational level with 48% of the sample having less than a college degree, 36.4% having a bachelor’s degree and 15.3% having a graduate degree. The sample was very skewed in terms of racial identity with 82.7% White, 1% Native American/American Indian, 1.4% Black, 1% Asian, 6.7% Hispanic/Latino/x and 7.2% multiracial.

### Self-report measures

#### Early adversity measures

##### *Perceived childhood SES*^73^

In an attempt to measure adversity as experienced in childhood we measured SES in childhood retrospectively. We assessed childhood SES by summing three individual yes/no (yes = 1, no = 0) items (e.g., “I grew up in a relatively wealthy neighborhood”) (*α* = 0.684).

##### Perceived unpredictability

Using methods adapted from Mittal et al.^74^ and Szepsenwol et al.^75^ participants responded to five five-point likert scale items with one item reverse coded (0—never true, 1—rarely true, 2—sometimes true, 3—often true, 4—very often true) assessing their family functioning prior to age 16 (e.g., prior to age 16 “things were pretty calm and stable in my house”; “people moved in and out of my house a lot”). We summed and averaged these six items (*α* = 0.828).

##### Sexual abuse

To assess sexual abuse, participants reported if they had experienced sexual assault prior to the age of 12 (0—no, 1—yes). The specific wording of the question was as follows: “Before the age of 12, I believe that I have been sexually abused by someone.”

##### Non-sexual abuse: (ACES)^76^

We utilize three individual items from the ACES scale to address non sexual abuse and summed across items (i.e., “swear at you, insult you”, “push, grab, slap or throw something at you”, “No one in your family loved you or thought you were important”) (*α* = 0.758).

#### Sexual behavior

##### Sexual partner number

Participants reported the number of male and female sexual partners they have had across their lifetime. To account for skewness, we took the natural log of this variable for both male and female partners.

##### Sexual debut

Participants reported the age at which they had their first sexual experience with a man or woman (i.e., “What was the age of your first significant sexual contact with a woman?”). We then took the younger of the two ages to create a measure of “earliest debut.”

##### Safe sex behavior questionnaire (SBSQ)^77^

This 27-item scale assesses participants’ engagement in safe sex behaviors. A few items are exclusionary to SGD populations. To validly reduce the scale and exclude such question we conducted an exploratory factor analysis retaining any questions that had factor loadings at or greater than 0.5 in the first component. This analysis resulted in a much shorter scale (8 items). Responses range from 1 (never) to 4 (always). We then summed the eight items as our final measure (*α* = 0.866).

#### Self-reported sexual reward sensitivity

##### Sexual reward responsiveness subscale

To address sexual reward responsiveness, participants responded to five questions adapted from the reward responsiveness subscale of the Behavioral Avoidance/Inhibition scale (BIS/BAS)^78^. Using the same Likert scale, we modified each question to be sex specific (e.g., “When I see an opportunity for sex with someone I find attractive I get excited right away”). We then summed the five questions to create one score of sexual reward sensitivity (*α* = 0.688). Given that this measure is new we checked for validity in measuring our construct. After conducting a confirmatory factor analysis along with examining correlations among related study variables we concluded that the five-item measure of sexual reward sensitivity is valid and indexes an individual’s sensitivity to the potential for sexual rewards and sexual activity. Please see supplementary materials “Construct validity for self reported sexual reward sensitivity” for further information.

#### Auxiliary variables

##### Sexual attraction past 12 months

Participants reported their degree of sexual attraction to both men and women, 1 (zero sexual attraction) to 5 (high sexual attraction).

*Impulsivity*^79^.

*Parental care quality* prior to age 16.

See Supplementary materials “Auxiliary variables” for more information.

### Neurobiological sexual reward sensitivity task

The EEG task used in the present study (called the “Sexy Doors Task”) was designed as a modification of the Doors Task commonly used in the EEG literature to assess reward sensitivity^60^. See Supplementary Materials section “Sexy doors task” for more information regarding the design of the task and its relationship to the classic Doors Task.

In the Sexy Doors Task, participants were presented with two doors on a computer screen and instructed to select a door. After selecting a door, a fixation cross appeared on the screen for 500 ms, followed by either a sexual image (e.g., image of two individuals engaging in sex with one another) or a neutral image (e.g., non-affective image such as a towel or a toaster). Sexual images were matched to the participant’s preference, such that prior to the task, participants chose to see either women engaging in sex with other women, men engaging in sex with women, or a mix of both. The image remained on the screen for 2000 ms, after which the participant was presented with the two doors again. The Sexy Doors Task had a total of 200 trials (100 sexual images, 100 neutral images) divided into 4 blocks. See Supplementary Materials “Image selection and presentation” for additional information about the images used.

### EEG procedures

Participants included in the EEG portion of data collection (N = 65) were fit with a 32-channel, active electrode cap manufactured by BrainVision (Morrisville, NC). Electrodes were arranged according to the 10–20 System^80^ and all impedances were kept below 10 kΩ. Data were recorded with a 500 Hz online sampling rate, a left mastoid reference (TP9), and a ground electrode in the middle of the forehead (FPz). Electrode FP2 situated above the right eyebrow was used to record eye-movements.

EEG data were processed in MATLAB with the EEGLAB^81^ and ERPLAB^82^ toolboxes. Data were downsampled to 250 Hz, bandpass filtered from 0.1 to 30 Hz, and epoched from − 200 to 1000 ms relative to the onset of the feedback image. The average voltage in the 200 ms window preceding image presentation served as the baseline. Eye movement artifacts were corrected for using eye movement correction procedure (EMCP)^83^, with an average trial loss of less than 2% across all files.

Artifact-free epochs were binned and averaged by image type (sexual vs. neutral), and each participant was then left with an event-related potential (ERP) waveform that represented their neural response to sexual images and a waveform that represented their neural response to neutral images. We then subtracted the neutral waveform from the sexual waveform to create a difference waveform, consistent with prior RewP studies. We numerically quantified neural response to sexual stimuli by extracting the mean amplitude of this difference waveform at electrode FCz from 200 to 400 ms post-image presentation, consistent with prior RewP research^60,84,85^ using the ERP Measurement Tool in ERPLab. Two participants were excluded from the EEG analysis due to equipment malfunctions during data collection, resulting in a final sample size of 63 participants.

#### Image arousal

Each participant who took part in the EEG portion of the study reported on how arousing they found the sexual images during the Sexy Doors Task (“How arousing did you find the images in the task”) with a scale of 1–10 (1 not arousing at all—10 extremely arousing). Mean arousal rating for images across the entire task suggests that the images were acceptable for use (M = 5.83).

#### Image type

The sexual images utilized in this study were obtained from the Concordia sexual image database^86^ and the neutral images were non-affective images obtained from the International Affective Picture System (IAPS). Importantly, the sexual images presented were matched to each participants’ preference such that prior to starting the task, participants elected to see images of women engaging in sex with other women, men engaging in sex with women, or a mix of both. Image choice was skewed with 78% of EEG participants choosing mixed images, 14% chose to see men engaging in sex with women stimuli and 8% chose to see women engaging in sex with other women.

## Analytic plan

We ran multiple structural equation models (SEM) using the Lavaan package in R using the default maximum likelihood estimator to test our hypotheses. Given our planned missingness design, we also ran modern missing data techniques using full information maximum likelihood (FIML). Past work validates the use of FIML in planned missingness designs^87^. See Supplementary Materials “Analytic plan” for further information.

### Supplementary Information

Supplementary Information.

## Data Availability

Data will be made available upon reasonable request to the corresponding author.
