# Supplementary material for: Early adversity and sexual diversity: the importance of self-reported and neurobiological sexual reward sensitivity
Source: Sci Rep. 2024 Apr 15;14:8717. doi: 10.1038/s41598-024-58389-w (PMC11018754; doi:10.1038/s41598-024-58389-w)
Supplement: Supplementary file 1 — Supplementary Information. [file 41598_2024_58389_MOESM1_ESM.docx]

EARLY ADVERSITY AND SEXUAL DIVERSITY: THE IMPORTANCE OF

SELF-REPORTED AND NEUROBIOLOGICAL

SEXUAL REWARD SENSITIVITY

Jenna Alley^1^*, Amy Mcdonnell^2^, Lisa M. Diamond^2^

^1^Department of Psychiatry and Biobehavioral Sciences, University of California, Los Angeles, CA

^2^ Department of Psychology, University of Utah, Salt Lake City UT

**Supplementary materials**

**Planned missingness**

Planned missingness designs allow researchers to maximize power within study designs that have “high-value but high-expense” procedures, such as EEG. Planned missingness designs allow for far greater power than standard techniques such as listwise deletion (Little & Rhemtulla, 2013). One method of planned missingness, and the one used in the current manuscript, is a two-method design (Graham, Taylor et al., 2006; Little & Rhemtulla, 2013; Rhemtulla & Little, 2012). A two-method design is extremely useful when a researcher has two different measures of the same construct (Little & Rhemtulla, 2013), one which is relatively easy to obtain but prone to systematic error (e.g., self-report) and another which is more difficult and time-consuming to obtain but more reliable (e.g., biomarkers, fMRI, EEG etc.,). To use this method, the “easy-to-obtain” measure should be administered to the entire sample, using a sample size large enough to detect the proposed effect, while the “hard-to-obtain” measure is administered to a *random subsample* of participants (Rhemtulla & Little, 2012). Because the missing data is missing at random, modern missing data techniques such as multiple imputation and full information maximum likelihood (FIML) can be used to account for the missing data, allowing for greater statistical power in testing hypotheses. As used in the present manuscript, more recent missing data approaches such as FIML are quickly becoming the preferred method for planned missingness designs (elaborated in detail in the section “Analytic plan”) and better fit the current analysis than other techniques such as multiple imputation.

**EEG Supplementary Materials**

*Sexy Doors Task*

The Sexy Doors Task utilized in this study was designed as a modification of the classic Doors Task commonly utilized in the EEG literature to elicit the RewP (Proudfit, 2015). In the classic Doors Task, participants are presented with two doors on the screen and instructed to select a door. Behind one door they win money behind the other door they lose money. The “win” and “loss” conditions are typically depicted with either an upward-facing green arrow (win) or a downward-facing red arrow (loss). In this classic Doors Task, each trial begins with the presentation of a fixation cross, followed by the two doors on the screen, which remain until the participant selects a door. Following their choice, a participant is presented with another fixation cross, followed by the feedback stimulus of either a green or red arrow, representing either monetary gain or loss. The reward positivity is time-locked to the onset of the feedback stimulus.

In order to assess participant response to *sexual* reward rather than *monetary* reward, our modified, Sexy Doors Task followed this exact same procedure with the exception of the *type* of feedback given. Participants were first presented with a fixation cross for 500ms, followed by two doors on the screen, which remained until the participant selected a door. Following their choice, they were again presented with a fixation cross (500ms) followed by either a sexual image or a neutral image (instead of green or red arrows). There was no monetary gain or loss associated with this task, as the images themselves were meant to simulate reward (sexual image) and non-reward (neutral image). The RewP was time-locked to the onset of the feedback image. Participants completed 200 trials of this task divided into four blocks, with 100 sexual stimuli and 100 neutral stimuli presented to each participant. The task took approximately 33 minutes to complete.

*Image Selection and Presentation*

The sexual images utilized in this study were obtained from the Concordia sexual image database (Shilhan, 2017) and the neutral images were non-affective images obtained from the International Affective Picture System (IAPS). Importantly, the sexual images presented were matched to each participants’ preference such that prior to starting the task, participants elected to see images of women engaging in sex with other women, men engaging in sex with women, or a mix of both. This was done to ensure that the sexual images would actually be perceived as rewarding for that particular participant. Images were presented in color in the center of the screen for 2000ms per picture.

*Additional ERP Component Discussion*

As shown in previous work utilizing the classic Doors Task, we expected to observe a positive deflection in amplitude starting approximately 200ms following stimulus presentation in both reward and non-reward conditions, with a more positive deflection in response to the rewarding conditions (Holroyd et al., 2011; Levinson et al., 2017). In order to measure RewP, one would then subtract the positive loss amplitude from the positive reward amplitude to produce a “difference” wave; this “difference” score which represented the RewP distinguishes RewP from closely related components such as N200 (Holroyd et al., 2011). However, as shown in Figure 2, we observe a negative deflection in amplitude around 200ms for both sexual and neutral images. Although the polarity of this waveform is not as expected, the time course of the ERP and the ways in which the two conditions differ is in line with previous literature (Holroyd et al., 2011). Specifically, we see significant deflections in amplitude around 200 ms post stimulus and our reward condition (sexual images) was reliably more positive than neutral conditions. Holroyd et al. (2011) state that while taking the difference between these two conditions should help distinguish the RewP component from other ERPs within the same time window; some experimental parameters can result in RewP patterns that more closely resemble an N2 (Brown & Cavanagh, 2018, 2020; Brown et al., 2021); this may account for the patterns observed in the present work and reflects patterns in other scholars’ recent work utilizing RewP.

For example, Brown and Cavanagh (2018) found that novel pleasant images resulted in greater negative deflections than did images of benign and common stimuli, such as money and arrows. They suggested that the combination of novel highly pleasant/arousing images can result in waveforms that more resemble the ERP component N2. The possibility that highly pleasant/arousing and novel stimuli can result in “flipped” polarity of the RewP waveform is a distinct possibility in the present work, given that our stimuli were explicit images of individuals engaging in sex with one another. Further given that there is evidence that sexual experience can have an impact on ERPs when viewing sexual images (Prause et al., 2015) with the idea that individuals willing to sign up for sexuality studies generally have higher sexual experience and more positive sexual attitudes there is also a chance that our waveform is reflecting the increased sexual experience and comfort of our sample and therefore future work should consider measuring and controlling for such experiences such as porn usage and comfort with sexual imagery.

Based on extant published work, we conclude that the negative polarity of our RewP waveform may be due to the nature of our images (novel positive and complex which can impact processing (Dawson et al., 2019). Specifically reflecting work by Brown and Cavanagh (2018) we believe that the novelty and general surprising nature of our stimuli likely created large N2/FRN ERP , therefore overshadowing the superimposed RewP. However, given that the two conditions are significantly different (with reward conditions being significantly more positive than neutral conditions) we have chosen to retain our operationalization of neurobiological sexual reward sensitivity as the difference between the two conditions (i.e, subtracting the neutral amplitudes from the sexy images amplitudes).

**Analytic plan - FIML**

Zhang and Yu, (2021) show that FIML can be used to effectively account for planned missingness producing unbiased estimates, especially when the full sample is greater than 100. In essence the FIML function predicts missing data based on the present values and the pattern of missingness and estimates a likelihood function for each individual in the dataset producing estimates that reflect the full sample size of the dataset (Allison, 2003). Zhang and Yu (2021) state that when the missingness is closer to 80%, certain estimates may be slightly skewed, however this issue is far greater in studies with samples closer to 100 total participants. While the sample size of the current project (*n* = 208) should be adequate, it is important to remain mindful of the limitations of these techniques. According to Zhang and Yu (2021) since our sample is closer to 200 and missingness is approximately 80% for the EEG measurements, our fit indices may be skewed in a conservative direction, yielding higher RMSEA and lower CFI values. Similarly, their findings suggest that with the current design, there is a risk for slight inflation of regression estimates. Other missing data techniques (such as multiple imputation) have less empirical work demonstrating appropriateness for planned missingness designs. Further some work suggests that the use of FIML is more robust when accounting for missing data in SEM (Enders, 2001; Little et al., 2014). Finally, Enders (2008) provides evidence for the use of auxiliary variables when using FIML to improve estimates, and indicate that the most reliable practice is to include auxiliary variables when using FIML, even when some of the auxiliary variables themselves have missing values.

**Construct validity for self-reported sexual reward sensitivity**

Given that the scale used to index self-reported sexual reward sensitivity was designed to measure one construct “self reported sexual reward sensitivity” we conducted a confirmatory factor analysis. Using R with packages Lavaan, and Psych we ran a confirmatory factor analysis. This factor analysis revealed that the fit when using the original 5 items together (Item 1, “When I have good sex I love to keep doing it”; Item 2, “When I have sex with someone I want I feel excited and energized.”; Item 3, “When I see an opportunity for sex with someone I find attractive I get excited right away.”; Item 4, “When someone I like tries to have sex with me, it affects me strongly.”; Item 5, “It would excite me to have a new sexual partner.”) to create an average score for the scale is poor (*χ^2^*(5) = 34.496, *p* = .000, CFI = .854, RMSEA = .171, SRMR = .067), further when the reliability of this scale was fair (*α* = .688). The factor loadings for each item were as follows; Item 1 = .70, Item 2 = .63, Item 3 = .69, Item 4 = .49, Item 5 = .41. Given the low loadings of items 4 and 5 we then tested the reliability of a scale using only items 1-3 and it was poorer than the scale with all five items (*α* = .674). Given that item 5 had the lowest loading we dropped it and again calculated the reliability of the scale when only using items 1-4, the reliability was now at an acceptable level (*α* = .702). Because the reliability using these items is better than the 5 item and 3 item versions we examined factor loadings using a confirmatory factor analysis approach. The model fit was similar to the 5 item version of the scale (*χ^2^*(2) = 16.465, *p* = .000, CFI = .912, RMSEA = .190, SRMR = .058) and the factor loadings were as follows; Item 1 = .77, Item 2 = .68, Item 3 = .60, Item 4 = .44). Given that the factor loadings are acceptable with both the 4 and 5 item version for the scale coupled with the fact that the reliability only changes marginally with the 4 item version we have chosen to retain all items in the scale and use all five items to create an average score for sexual reward sensitivity.

We also examined how our newly designed scale correlated with other collected variables that weren’t used in our primary analysis and again found substantial evidence of validity. First, as outlined in the correlation table, conscious SRS doesn’t correlate with automatic SRS. This is neither expected or unexpected as we know very little about how different forms of SRS function. However it is important to point out that while conscious SRS is not correlated with automatic SRS (which is represented by the difference in mean amplitude across sexy and neutral conditions) it positively correlates with the mean amplitude when viewing only sexual images (*r =* .301, *p* = .047). Further the scale positively correlates with many single items from the perceived benefits section of the Cognitive Appraisal of Risky Events (Revised) (CARE-R; Katz, et al., 2000). The benefits sub scale of this measure assesses an individual's perceptions of potential benefits associated with various risky activities, including a wide range of sexual behaviors. Specifically simple zero order correlations show that conscious SRS positively correlates with many of the single item questions asking the likelihood (1- not likely at all, 7- extremely likely) of experiencing positive consequences (e.g., pleasure, feel good about yourself) (e.g., “sex with a regular partner” (*r =* .376, *p* < .001), “sex with someone I just met” (*r =* .392, *p* < .001), “sexual intercourse while under the influence of alcohol with someone I just met or do not know well” (*r =* .330, *p* < .001), “sex without a condom with someone I just met or do not know well” (*r =* .248, *p* < .001), “sex with a new partner” (*r =* .376, *p* < .001)). These relationships suggest that our measure of conscious SRS is reflecting perceptions of positive or rewarding outcomes of all sexual behavior including risky sex. Further we found that our scale did not significantly correlate with the general reward responsiveness subscale from the BIS/BAS (*r* = .129, *p* = .069). This again suggests that our measure of conscious SRS is in fact measuring a form of specific reward sensitivity as opposed to general rewards.

**Auxiliary Variables Detailed Information**

*Sexual Attraction:* Participants reported their degree of sexual attraction within the past 12 months to both men and women separately. They reported this on a five-point Likert scale ranging from 1 (zero sexual attraction) to 5 (high sexual attraction).

*Impulsivity:* This measure consists of 63 yes/no items meant to assess an individual's level of impulsiveness, venturesomeness and empathy (Eysenck & Eysenck, 1978). In order to isolate impulsivity, we conducted an exploratory factor analysis and identified 14 items that loaded on to a factor portraying impulsivity, with factor loadings greater than .5 (e.g., " Do you generally do and say things without stopping to think?", "Are you an impulsive person?", “Do you need to use a lot of self-control to keep out of trouble?”). The resulting scale has good reliability (*α* = .808).

*Parental Care Quality* Participants reported on the quality of care from both male and female care givers prior to age 16. Participants were instructed that if they did not grow up with biological parents to report care quality based on the primary care giver of each sex/gender. Participants reported on parental quality across 9 items (e.g., “She spoke to me with a warm and friendly tone”, “She insulted me or put me down”, “She made me feel I wasn’t wanted”). We then summed and averaged the scores for male and female caregivers separately and then summed those averages to create an overall parental quality score.

**Limitations and Future Directions**

As noted earlier, an important limitation to the present study is the inability to rule out the possibility that the relative *novelty* of sexual stimuli may complicate the EEG findings. Given the contemporary availability of sexual images on the internet, and potential associations between high-risk sexual behavior and pornography use, we cannot determine whether familiarity with sexual images played a role in our findings. Future work should seek more comprehensive assessments of individuals’ familiarity with sexual versus nonsexual visual stimuli, and should also consider the inclusion of measures designed to assess the level of novelty and or surprise individuals experience when exposed to rewarding stimuli.

Another limitation of the current work is that we only examine cisgender women. Although this methodological choice makes is easier for us to examine within-group variation in a novel construct (sexual reward sensitivity), future work should examine these dimensions in cisgender men, as well as gender-diverse individuals (notably, the latter group has disproportionately high exposure to childhood adversity, as reviewed by Diamond et al., (2021). The potential moderating role of neurodiversity in the processing of risks and rewards also warrants greater attention, as do other forms of diversity that intersect with early adversity (such as ethnic and racial marginalization). Another unique aspect of our sample is the fact that all of our participants lived in the Salt Lake City area of Utah. Although we were able to recruit a very sexually diverse sample, due to the predominant religious groups in the area, discussions surrounding pleasure, sexuality and porn are rarely covered in sexual education in Utah; this makes it especially difficult to know whether and how variability in respondents’ exposure to sexual information (and hence their degree of familiarity with sexual images) may have played a role in our findings.

An important direction for future research involves greater attention to a broader range of sexual risk behaviors than are typically examined in studies of life history strategies, such as same-gender behavior. Alley and Diamond (2021) argued that if early adversity does regulate an individual’s sensitivity to rewards sensitivity, then we need to devote greater attention to assessing a broader range of behaviors that might be augmented by such a process, such as non-reproductive sexual behaviors that are highly rewarding; this may include not only same-gender behavior, but solitary sexual behavior and other mechanisms for achieving sexual reward. Similarly, in addition to focusing on the health risks of “fast” life history behaviors (such as early sexual debut or sex without contraception), researchers should focus on the social risks of sexual behavior, such as stigma and marginalization (which accompany not only same-gender behavior, but other non-normative sexual practices).

Perhaps the most important direction for future research involves greater attention to *pleasure* as a core motivation for sexual behavior of all forms, regardless of their degree of risk. Treating pleasure, and individuals’ responsiveness to opportunities for pleasure, as *variables* rather than constants may yield superior approaches to sexual health education for both heterosexual and sexually diverse populations. When educating adolescents and adults with a history of adversity about safe sexual practices, it may be important to take account for the fact that they may experience sexual motivations *differently* from other populations, on both conscious and automatic levels. Instead of simply asking youths (from both adverse and non-adverse backgrounds) to ignore or suppress their sexual motivations (as is the case with abstinence-based programs, which are relatively ineffective, (Kirby, 2008; Kohler et al., 2008), our research suggests the importance of intervention approaches that forthrightly account for and address individual differences in sexual motivation and sexual reward responsiveness, and can empower youths from diverse backgrounds to actively assess and think through their own particular approach to “reward/risk” tradeoffs. Comprehensive sexual education programs should include discussion of sexual practices that are low in health risks but highly likely to result in sexual reward and pleasure. In fact, a meta-analysis conducted by Zaneva et al., (2022) shows that sexual health interventions which incorporate considerations of safety and pleasure significantly increase condom use and can actually have positive effects on knowledge-based attitudes surrounding sex.

**Additional Supplementary references**

Alley, J., & Diamond, L. M. (2021). Early childhood adversity and women’s sexual behavior: The role of sensitivity to sexual reward. *Developmental Review*, *61*, 100982.

Allison, P. D. (2003). Missing data techniques for structural equation modeling. *Journal of abnormal psychology*, *112*(4), 545.

Brown, D. R., & Cavanagh, J. F. (2020). Novel rewards occlude the reward positivity, and what to do about it. *Biological Psychology*, *151*, 107841.

Brown, D. R., Jackson, T. C., & Cavanagh, J. F. (2021). The reward positivity is sensitive to affective liking. *Cognitive, Affective, & Behavioral Neuroscience*, 1-10.

Dawson, S. J., Huberman, J. S., Bouchard, K. N., McInnis, M. K., Pukall, C. F., & Chivers, M. L. (2019). Effects of individual difference variables, gender, and exclusivity of sexual attraction on volunteer bias in sexuality research. *Archives of Sexual Behavior*, *48*, 2403-2417.

Diamond, L. M., Dehlin, A. J., & Alley, J. (2021). Systemic inflammation as a driver of health disparities among sexually-diverse and gender-diverse individuals. *Psychoneuroendocrinology*, *129*, 105215.

Enders, C. K. (2001). The impact of nonnormality on full information maximum-likelihood estimation for structural equation models with missing data. *Psychological methods*, *6*(4), 352.

Enders, C. K. (2008). A note on the use of missing auxiliary variables in full information maximum likelihood-based structural equation models. *Structural Equation Modeling: A Multidisciplinary Journal*, *15*(3), 434-448.

Eysenck, S. B., & Eysenck, H. J. (1978). Impulsiveness and venturesomeness: Their position in a dimensional system of personality description. *Psychological reports*, *43*(3_suppl), 1247-1255.

Graham, J. W., Taylor, B. J., Olchowski, A. E., & Cumsille, P. E. (2006). Planned missing data designs in psychological research. *Psychological methods*, *11*(4), 323.

Holroyd, C. B., Krigolson, O. E., & Lee, S. (2011). Reward positivity elicited by predictive cues. *Neuroreport*, *22*(5), 249-252.

Levinson, A. R., Speed, B. C., Infantolino, Z. P., & Hajcak, G. (2017). Reliability of the electrocortical response to gains and losses in the doors task. *Psychophysiology*, *54*(4), 601-607.

Katz, E. C., Fromme, K., & D'amico, E. J. (2000). Effects of outcome expectancies and personality on young adults' illicit drug use, heavy drinking, and risky sexual behavior. *Cognitive Therapy and Research*, *24*(1), 1-22.

Kirby, D. B. (2008). The impact of abstinence and comprehensive sex and STD/HIV education programs on adolescent sexual behavior. *Sexuality Research & Social Policy*, *5*(3), 18. doi:10.1525/srsp.2008.5.3.18

Kohler, P. K., Manhart, L. E., & Lafferty, W. E. (2008). Abstinence-only and comprehensive sex education and the initiation of sexual activity and teen pregnancy. *Journal of adolescent Health*, *42*(4), 344-351.

Little, T. D., Jorgensen, T. D., Lang, K. M., & Moore, E. W. G. (2014). On the joys of missing data. *Journal of pediatric psychology*, *39*(2), 151-162

Little, T. D., & Rhemtulla, M. (2013). Planned missing data designs for developmental researchers. *Child Development Perspectives*, *7*(4), 199-204.

Proudfit, G. H. (2015). The reward positivity: From basic research on reward to a biomarker for depression. *Psychophysiology*, *52*(4), 449-459.

Rhemtulla, M., & Little, T. D. (2012). Planned missing data designs for research in cognitive development. *Journal of Cognition and Development*, *13*(4), 425-438.

Shilhan, J. (2017). *Attentional Processing of Visual Sexual Stimuli and the Concordia Sexual Image Dataset* (Doctoral dissertation, Concordia University).

Zaneva, M., Philpott, A., Singh, A., Larsson, G., & Gonsalves, L. (2022). What is the added value of incorporating pleasure in sexual health interventions? A systematic review and meta-analysis. *Plos one*, *17*(2), e0261034.

Zhang, C., & Yu, M. C. (2021). Planned Missingness: How to and How Much?. *Organizational Research Methods*, 10944281211016534
